# Explaining ethnic differentials in COVID-19 mortality: cohort study

**DOI:** 10.1093/aje/kwab237

**Published:** 2021-09-29

**Authors:** G David Batty, Bamba Gaye, Catharine R Gale, Mark Hamer, Camille Lassale

**Affiliations:** 1 Department of Epidemiology and Public Health, University College London, UK; 2 Paris Cardiovascular Research Center-INSERM U970, France; 3 MRC Lifecourse Epidemiology Unit, University of Southampton, UK, Lothian Birth Cohorts, Department of Psychology, University of Edinburgh, UK; 4 Division of Surgery and Interventional Sciences, University College London, London; 5 Hospital del Mar Medical Research Institute, Barcelona, Spain, CIBER of Pathophysiology of Obesity and Nutrition, Madrid, Spain

**Keywords:** ethnicity, COVID-19, cohort study, UK Biobank

## Abstract

Ethnic inequalities in coronavirus disease 2019 (COVID-19) hospitalizations and mortality have been widely reported but there is scant understanding of how they are embodied. The UK Biobank prospective cohort study comprises around half a million people who were aged 40-69 years at study induction between 2006 and 2010 when information on ethnic background and potential explanatory factors was captured. Study members were prospectively linked to a national mortality registry. In an analytical sample of 448,664 individuals (248,820 women), 705 deaths were ascribed to COVID-19 between 5^th^ March, 2020 and 24^th^ January, 2021. In age- and sex-adjusted analyses, relative to White participants, Black study members experienced around five times the risk of COVID-19 mortality (odds ratio; 95% confidence interval: 4.81; 3.28, 7.05), while there was a doubling in the South Asian group (2.05; 1.30, 3.25). Controlling for baseline comorbidities, social factors (including socioeconomic circumstances), and lifestyle indices attenuated this risk differential by 34% in Black study members (2.84; 1.91, 4.23) and 37% in South Asian individuals (1.57; 0.97, 2.55). The residual risk of COVID-19 deaths in ethnic minority groups may be ascribed to a range of unmeasured characteristics and requires further exploration.

*Abbreviations:* coronavirus disease 2019 (COVID-19)

ORIGINAL UNEDITED MANUSCRIPT

## Introduction

Although the 2009 swine influenza (H1N1) pandemic did not have the acute and far-reaching societal and economic impact of coronavirus disease 2019 (COVID-19), severe cases were nonetheless characterised by ethnic disparities.[1-3] In the present pandemic, there is now abundant evidence from the US and the UK of such differentials whereby, relative to White individuals, people of African-Caribbean (Black), Latinx, and, South Asian origin, experience the greatest burden of severe acute respiratory syndrome coronavirus 2 (SARS-CoV-2) – the infection that causes COVID-19 – and hospitalization for, and mortality from, the disease.[4,5]

Understanding how these ethnic variations in COVID-19 are embodied is central to the process of disease prevention. Individuals from different ethnic backgrounds differ in health behaviours, body composition, comorbidities, immune profiles, and socioeconomic circumstances, amongst other characteristics.[6] The best evidence of ethnic differentials in COVID-19 largely draws on observational studies generated from electronic health records where potential explanatory or mediating factors, aside from socioeconomic status and somatic morbidities, are rarely captured.[4,5] Thus, the role of mental health,[7] lifestyle factors (e.g., body mass index, alcohol intake),[8] and physiological indices (e.g., systemic inflammation)[9,10] is untested in this context.

Using data from UK Biobank, a field-based prospective cohort study, we have shown that people of South Asian and particularly African-Caribbean (Black) heritage experienced a markedly elevated risk of a diagnosis of severe COVID-19, and up to half of these differentials was explained by socioeconomic status and lifestyle indices.[11] In that study, hospitalisation for COVID-19 was the outcome of interest. As the pandemic has unfolded, a sufficiently high number of deaths from the disease have accumulated in this cohort to allow us to test these original results with new data.

ORIGINAL UNEDITED MANUSCRIPT

## Methods

UK Biobank is a prospective cohort study, the sampling and procedures of which have been well described.[12,13] Baseline data collection took place between 2006 and 2010 across twenty two research assessment centres in the UK giving rise to a sample of 502,655 people aged 40 to 69 years (response 5.5%). Ethical approval was granted by the North-West Multi-centre Research Ethics Committee, and the research was carried out in accordance with the Declaration of Helsinki of the World Medical Association; participants gave written consent.

*Assessment of ethnicity*

Data on ethnicity and all covariates used in the present analyses were collected at baseline. Using similar enquiries to those from the 2001[14] and 2011[15] UK census, ethnicity was self-classified as White (British, Irish, any other White background); Asian or Asian British (Indian, Pakistani, Bangladeshi, any other South Asian background); Black or Black British (Caribbean, African, any other Black background); Mixed; Chinese; or 'other'.[11] With a low number of COVID-19 deaths occurring in the latter three categories owing to the low denominator, these were collapsed into a single 'other' group.

*Assessment of covariates*

In the present study, we included those covariates shown to be associated with COVID-19 hospitalisation in prior analyses.[11] Individual socioeconomic status was captured using educational qualifications (university degree, other qualifications, no qualifications). Occupational classification was also available but in a subgroup of participants and based on current job from which we derived two categories: non-manual (managerial positions, technical, administrative) and manual (sales and customer service, process, plant & machine operatives). The Townsend index of neighbourhood deprivation, a group-level indicator of poverty, is based on national census data, with each participant assigned a score corresponding to the postcode of home address; higher values denote greater disadvantage. The number of people in the household of the study member was also recorded (living alone, 2 people, 3 people, 4 people or more).[16] Levels of cigarette smoking (never, former, current) and alcohol consumption (never, special occasions, 1-

ORIGINAL UNEDITED MANUSCRIPT

3/month, 1-2 week, 3-4/week, daily) were assessed using standard enquiries, and height, weight, and circumferences of waist and hip were measured using standard protocols.[17] Vascular or heart problems, diabetes, and chronic bronchitis, were based on self-reported physician diagnosis and hypertension was defined as systolic/diastolic blood pressure ≥ 140/90 mmHg and/or self-reported use of antihypertensive medication.[10] Study members were also asked whether they had ever been under the care of a psychiatrist for any mental health problem.[7] Available for a subgroup, white blood cell count (a marker of inflammation), glycated haemoglobin, and high-density lipoprotein cholesterol concentrations were based on assays of non-fasting venous blood.

*Ascertainment of mortality ascribed to COVID-19*

Participants were linked to long-standing national mortality records from which death from COVID-19, our outcome of interest, was denoted by the emergency International Classification of Disease (version 10) code U07.1 (COVID-19, virus identified). Deaths were ascertained between 5th March, 2020, and the end of follow-up on 24th January 2021.

*Statistical analyses*

To summarise the association of mortality with ethnicity we used logistic regression to compute odds ratios with accompanying 95% confidence intervals. With COVID-19 deaths occurring over a short period and being rare in the present study, odds ratios very closely resemble hazard ratios as computed using Cox regression analyses. We initially provide age- and sex-adjusted odds ratios; the most basic model and therefore our comparator. We then explored the impact of controlling for individual covariates by making separate (non-accumulative) adjustment for social factors, lifestyle factors, comorbidities, and biomarkers. Percentage change in effect estimates following statistical control was calculated as: $100*(\beta_{\text{complex adjustment}} - \beta_{\text{basic adjustment}})/ \beta_{\text{basic adjustment}}$ where basic adjustment was control for age and sex only, and complex adjustment was the addition of further covariates.[18] We also present results where all covariates were imputed using chain equations.

ORIGINAL UNEDITED MANUSCRIPT

## Results

Our analytical sample comprised 448,664 individuals (248,820 women). Compared with White study members, at baseline, people from the ethnic minority groups were slightly younger, and markedly more likely to live in higher occupancy households, reside in poorer neighbourhoods, work in manual occupations, and have diabetes (Web Table 1). People from South Asian and other backgrounds were, however, more likely to have experience of higher education than White and Black individuals. Black people had amongst the lower prevalence of chronic bronchitis and mental health problems but the highest burden of hypertension.

Mortality surveillance in the analytical sample gave rise to 705 deaths from COVID-19 (650 in White participants; 28 in Blacks; 19 in South Asians; and 8 in those from other ethnic groups). In table 1, we show the age- and sex-adjusted relation of the above covariates plus ethnicity to the risk of death from COVID-19. Unfavourable levels of all eighteen covariates were related to a higher risk of death from COVID-19 in minimally-adjusted analyses; only the point estimate for chronic bronchitis, while elevated, did not achieve statistical significance at conventional levels. For instance, there was a raised risk of COVID-19 death in people from disadvantaged socioeconomic background, those living alone, those with extant illness at baseline and those with a higher white blood cell count. While people with less healthy lifestyle choices typically experienced higher risk, the daily consumption of alcohol seemed to confer some protection.

As depicted in table 1 and figure 1, relative to White participants, Black study members experienced around five times the risk of COVID-19 mortality (age- and sex-adjusted odds ratio; 95% confidence interval: 4.81; 3.28, 7.05), while there was around a doubling in the South Asian group (2.05; 1.30, 3.25). There was evidence of a lack of precision in some of these analyses as evidenced by the breadth of the confidence intervals. We explored the impact of individual covariates by making separate (non-accumulative) adjustment for social factors, lifestyle factors, and comorbidities (figure 1). In Black participants, relative to the regression coefficients in the age- and sex-adjusted analyses, in separate adjustment, we found that social factors had the largest impact (3.12; 2.10, 4.61; 28% attenuation), whereas in people of South Asian

ORIGINAL UNEDITED MANUSCRIPT

backgrounds, it was comorbidities (1.55; 0.97, 2.46; 39% attenuation). Collectively, these covariates accounted for around one third of the disparity in COVID-19 deaths for Black individuals (2.84; 1.91, 4.23; 34% attenuation) as they did for South Asian study members (1.57; 0.97, 2.55; 37% attenuation).

That despite statistical control for an array of variables there remained a marked residual risk of death from COVID-19 in ethnic minority groups implicates other risk indices. Biological indices including high density lipoprotein cholesterol, glycated haemoglobin, and white blood cell count, associated with COVID-19 deaths in the present dataset, were available for 358,820 people in whom there were 578 COVID-19 deaths. Adding these variables to the comparator model yielded marked attenuation (1.35; 0.79, 2.32; 54% attenuation) for South Asian study members but not for Black individuals (4.69; 2.93, 7.53; 2% attenuation) (Web Table 2).

It is plausible that people from ethnic minority groups are more likely to be in service industry employment which requires them to have a person- or patient-facing role so potentially placing them at elevated risk of infection. In analyses of the subgroup with data on job title (N=322,353) there was a total of 328 deaths (Web Table 3). The original raised risk in Black individuals in the main analyses after adjustment for social factors (3.12; 2.10, 4.61) was elevated slightly when occupation was added to the model (3.96; 2.46, 6.36); again, statistical precision was modest owing to the small numbers of COVID-19 fatalities in this and other minority groups. Lastly, on imputing covariates (Web Table 4), a similar pattern of association was observed to that apparent in the main analyses, with an age- and sex-adjusted odds ratio for Black individuals of 3.99 (2.74, 5.80), which was attenuated by 31% after multiple adjustment which included occupation and biomarkers, to 2.58 (1.74, 3.84). In corresponding analyses for people from South Asian background, attenuation after the same statistical control was 47%.

**Discussion**

Our main finding was that, despite statistical control for social factors, lifestyle indices, biological factors, and comorbidities, there remained a markedly raised risk of COVID-19 mortality in people of African-

ORIGINAL UNEDITED MANUSCRIPT

Caribbean and South Asian origin in the UK. That we were able to replicate known associations with COVID-19 mortality for socioeconomic circumstances, comorbidities, age, and sex apparent in studies from the US,[19] UK,[20] Italy,[21] China,[22] and Brazil[23] gives us some confidence in the more novel results presented here for ethnicity.

The marked post-adjustment excess risk of COVID-19 in Black and South Asian study members suggests that unmeasured and/or unknown risk factors have a role.[6] While the present dataset is reasonably well-characterised for environmental factors, we do not have data on, for instance, life course socioeconomic position nor racial discrimination. Although understudied, racial discrimination appears to have an influence on selected health outcomes, most consistently mental health[24] and, of more relevance to the present study, respiratory conditions such as adult-onset asthma.[25] While vigorously advanced in some quarters as having a causative role in the current pandemic,[26-29] to the best of our knowledge, such links are untested empirically, rendering moot its role.

*Comparison with existing studies*

Although less well examined owing to it lower impact relative to the present pandemic, H1N1 revealed similar ethnic differentials to those reported herein [1,2] The Spanish influenza of 1918 was perhaps an exception: rates of hospitalisations and death were in fact seemingly lower in people of Black ethnic origin relative to Whites in the US[30] – the only year in the 20th century when being of Black origin appeared to confer some protection against death from influenza. In the current pandemic, the present findings of ethnic disparities are supported by observations made on populations from the US and the UK.[4,5] As discussed, while in-depth examination of the causes of these inequalities is rare owing to an absence of higher resolution data in most studies, effects seem to survive adjustment for extant morbidity and, when available, markers of poverty. [4,5 31-34] Partial attention by comorbidity, which for the first time to our knowledge featured mental illness,[7] was also seen herein, with a further diminution in risk offered by lifestyle and social factors which confirms our earlier work on hospitalisations for the disease.[11] Unlike the present analyses featuring death as the outcome of interest, in that study[11] we used a record of a positive

ORIGINAL UNEDITED MANUSCRIPT

in-patient test for COVID-19 as our outcome of interest. While this was assumed to be an indicator of disease severity – only serious cases are hospitalised in the UK which operates under a single, national health service – it is nonetheless likely that, after routine hospital-wide testing, some patients being treated for unrelated conditions were positive but asymptomatic for COVID-19. Our results here for death from the disease corroborates these earlier findings, however.[11]

*Study strengths and weaknesses*

The strengths of the study include the well-characterised nature of the study members and the full coverage of the population for cause of death from COVID-19. Our work is of course not without its weaknesses. Although the present cohort is large, there were too few deaths in selected ethnic groups – people from East Asian or mixed backgrounds, for instance – to facilitate analyses. Also, while ethnicity itself is stable over-time – UK data reveal that only 4% of census participants chose a different ethnic group a decade after their first declaration[35] – other baseline data are more likely to be time-varying in the period between study induction in UK Biobank and the present pandemic, in particular for comorbidity. This is a perennial issue in cohort studies and one we were able to investigate using data from a resurvey that took place around 8 years after baseline examination in a sub-sample of around 30,000 people. Analyses revealed moderate to high stability for some covariates, including education (r=0.86, p<0.001) and body mass index (r=0.90, p<0.001), whereas the magnitude was somewhat lower for diabetes (r=0.63, P<0.001) serious mental illness (r=0.64, p<0.001), and cigarette smoking (r=0.60, p<0.001, N=31037).

*Generalisability of the present findings*

With the present sample not being representative of the general UK population, death rates from leading causes and the prevalence of reported risk factors are known to be underestimates of those apparent in less select groups;[13] the same is likely to be the case for COVID-19 cases. This notwithstanding, for the following reasons, there is evidence that risk factor associations, including those presented herein for ethnicity, are externally valid.[13] First, the ethnic distribution in UK Biobank is similar to the UK 2001 and 2011 census data (Web Table 5). Second, relative to White Europeans, we found that South Asians have a

ORIGINAL UNEDITED MANUSCRIPT

markedly higher prevalence of diabetes and less favourable waist-to-hip ratio, whereas the greatest burden of hypertension was in Black and White participants (Web Table 1). These observations have been made across multiple studies.[36-38] Third, consistently higher rates of coronary heart disease in South Asians (the reverse in Black individuals), and a lower risk of cancer have been reported.[36,38-40] In analyses of data from the present study, we found this pattern of association (Web Table 6). Lastly, as described, in keeping with systematic reviews of ethnicity and COVID-19,[4,5] we have shown an increased risk of hospitalisations for COVID-19 among minority groups in the UK.[11] Taken together then, we regard the present results from UK Biobank to be generalisable.

In conclusion, in this well-characterised prospective cohort study, based on conventional risk factors, we were only able to partially understand how ethnic disparities in COVID-19 were embodied. Subsequent research should target additional factors uncaptured herein, including life course socioeconomic position and racial discrimination.

ORIGINAL UNEDITED MANUSCRIPT

**Table 1. Age- and sex-adjusted odds ratios for the association of baseline covariates and ethnicity (2006-10) with COVID-19 mortality (2020-21)**

| Baseline characteristic | Total No. | No. of Deaths | Odds ratio[a] | 95% CI | p-value |
|---|---|---|---|---|---|
| Ethnicity | | | | | |
| White | 426,265 | 650 | 1.00 | referent | |
| Black | 6,816 | 28 | 4.81 | 3.28, 7.05 | <0.001 |
| South Asian | 7,839 | 19 | 2.05 | 1.30, 3.25 | 0.002 |
| Other | 7,774 | 8 | 1.19 | 0.59, 2.40 | 0.63 |
| Demographic factors | | | | | |
| Age (per 1 year increase) | | | 1.15 | 1.14, 1.17 | <0.001 |
| Male (male vs. female) | | | 2.27 | 1.94, 2.65 | <0.001 |
| Social factors | | | | | |
| Education (high school vs. university) | | | 1.92 | 1.58, 2.33 | <0.001 |
| Occupation (manual vs. non-manual) | | | 1.99 | 1.57, 2.52 | <0.001 |
| Household size (living alone vs. 2 people) | | | 1.98 | 1.67, 2.35 | <0.001 |
| Area-based deprivation index (quintile 5 [highest] vs. 1) | | | 2.87 | 2.28, 3.62 | <0.001 |
| Lifestyle factors | | | | | |
| Alcohol (never vs. daily) | | | 2.78 | 2.14, 3.61 | <0.001 |
| Cigarette smoking (current vs. never) | | | 2.25 | 1.78, 2.85 | <0.001 |
| Body mass index[b] (per 1 $kg/m^2$ increase) | | | 1.11 | 1.09, 1.12 | <0.001 |
| Waist to hip ratio (per 0.1 increase) | | | 2.03 | 1.84, 2.24 | <0.001 |
| Co-morbidities | | | | | |
| Hypertension (yes vs. no) | | | 1.58 | 1.31, 1.91 | <0.001 |
| Cardiovascular disease (yes vs. no) | | | 2.25 | 1.85, 2.73 | <0.001 |
| Chronic bronchitis (yes vs. no) | | | 1.48 | 0.93, 2.33 | 0.10 |
| Diabetes (yes vs. no) | | | 3.15 | 2.60, 3.82 | <0.001 |
| Consultation with a psychiatrist (yes vs. no) | | | 1.60 | 1.30, 1.96 | <0.001 |
| Biomarkers | | | | | |
| White blood cell count (per 1 log $10^9$/L increase) | | | 3.26 | 2.50, 4.25 | <0.001 |
| High-density lipoprotein (per 1 mmol/L increase) | | | 0.43 | 0.33, 0.56 | <0.001 |
| HbA1C (per 1 log mmol/mol increase) | | | 7.75 | 5.51, 10.90 | <0.001 |

HbA1C, glycated hemoglobin.

[a] Odds ratios are expressed per category, or per SD increase for continuous variables. Analyses for occupational classification (N=322,353) and biomarkers (N=358,820) are based on subgroups of study members. All other analyses are based on the full cohort (N=448,664). Odds ratios for age and sex are mutually adjusted.

[b]Weight (kg)/height $(m)^2$.

ORIGINAL UNEDITED MANUSCRIPT

**Figure 1. Odds ratios for the association between ethnicity (2006-10) and COVID-19 mortality (2020-21)**

Covariates included in each model correspond to those described in table 1. For the Black group, attenuation of regression coefficients was: 28% after controlling for social factors; 17% for lifestyle; 10% for comorbidity; and 34% for all covariates combined. For the South Asian group: 4% after controlling for social factors; 30% for lifestyle; 39% for comorbidities; and 37% for all covariates combined. For the 'other' ethnic group: 91% after controlling for social factors; 108% for lifestyle; 66% for comorbidities; and 154% for all covariates combined.

ORIGINAL UNEDITED MANUSCRIPT

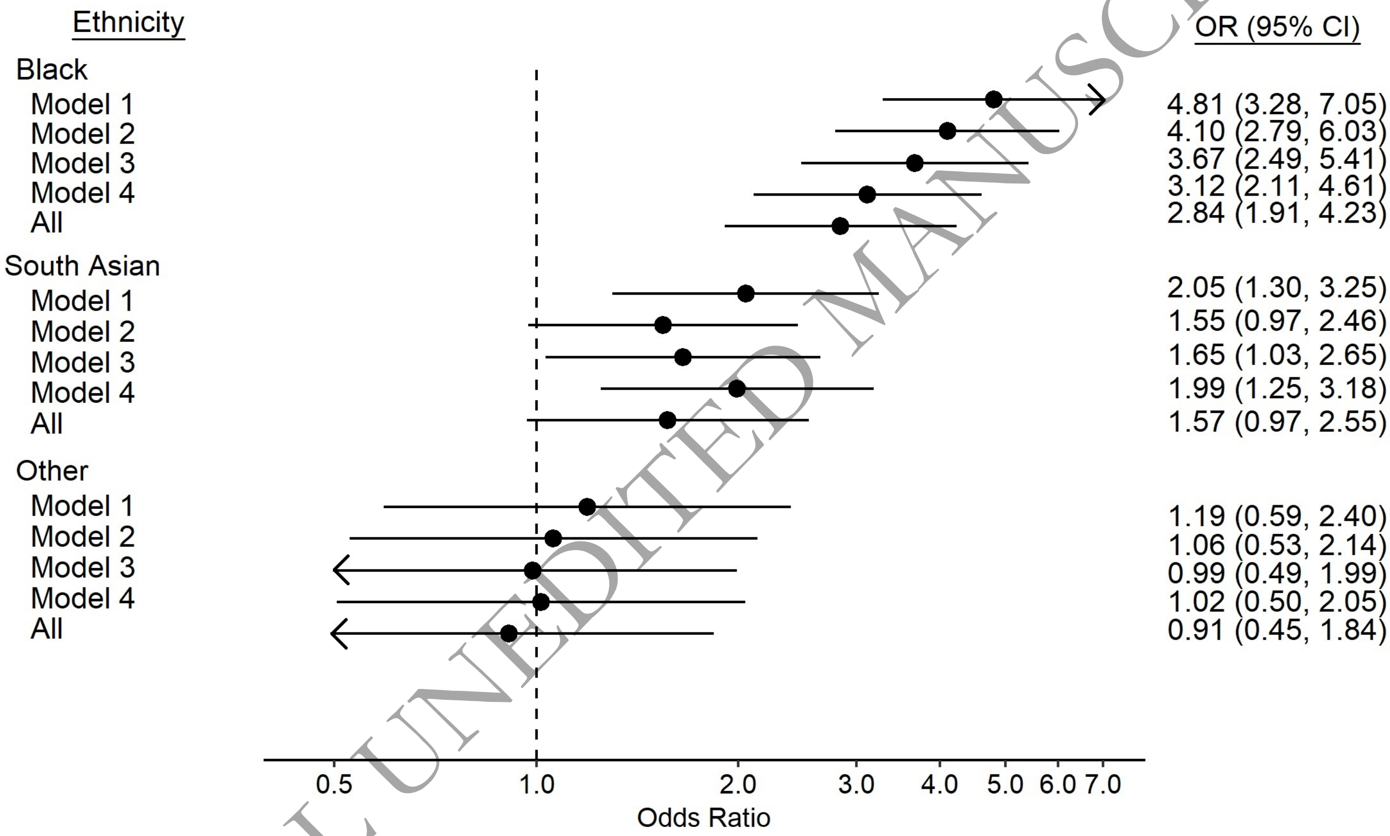

Ethnicity
OR (95% CI)
Black
Model 1
Model 2
Model 3
Model 4
All
4.81 (3.28, 7.05)
4.10 (2.79, 6.03)
3.67 (2.49, 5.41)
3.12 (2.11, 4.61)
2.84 (1.91, 4.23)
South Asian
Model 1
Model 2
Model 3
Model 4
All
2.05 (1.30, 3.25)
1.55 (0.97, 2.46)
1.65 (1.03, 2.65)
1.99 (1.25, 3.18)
1.57 (0.97, 2.55)
Other
Model 1
Model 2
Model 3
Model 4
All
1.19 (0.59, 2.40)
1.06 (0.53, 2.14)
0.99 (0.49, 1.99)
1.02 (0.50, 2.05)
0.91 (0.45, 1.84)
0.5
1.0
2.0
3.0
4.0
5.0
6.0
7.0
Odds Ratio

## Supplementary Material

Web_Material_kwab237

