# Explaining Ethnic Differentials in COVID-19 Mortality: A Cohort Study

**DOI:** 10.1093/aje/kwab237

**Published:** 2021-09-29

**Authors:** G David Batty, Bamba Gaye, Catharine R Gale, Mark Hamer, Camille Lassale

**Keywords:** cohort study, COVID-19, ethnicity, UK Biobank

## Abstract

Ethnic inequalities in coronavirus disease 2019 (COVID-19) hospitalizations and mortality have been widely reported, but there is scant understanding of how they are embodied. The UK Biobank prospective cohort study comprises approximately half a million people who were aged 40–69 years at study induction, between 2006 and 2010, when information on ethnic background and potential explanatory factors was captured. Study members were prospectively linked to a national mortality registry. In an analytical sample of 448,664 individuals (248,820 women), 705 deaths were ascribed to COVID-19 between March 5, 2020, and January 24, 2021. In age- and sex-adjusted analyses, relative to White participants, Black study members experienced approximately 5 times the risk of COVID-19 mortality (odds ratio (OR) = 4.81, 95% confidence interval (CI): 3.28, 7.05), while there was a doubling in the South Asian group (OR = 2.05, 95% CI: 1.30, 3.25). Controlling for baseline comorbidities, social factors (including socioeconomic circumstances), and lifestyle indices attenuated this risk differential by 34% in Black study members (OR = 2.84, 95% CI: 1.91, 4.23) and 37% in South Asian individuals (OR = 1.57, 95% CI: 0.97, 2.55). The residual risk of COVID-19 deaths in ethnic minority groups may be ascribed to a range of unmeasured characteristics and requires further exploration.

## Abbreviations:

CI: confidence interval
COVID-19: coronavirus disease 2019
OR: odds ratio

Although the 2009 swine influenza (H1N1) pandemic did not have the acute and far-reaching societal and economic impact of coronavirus disease 2019 (COVID-19), severe cases were nonetheless characterized by ethnic disparities (1–3). In the present pandemic, there is now abundant evidence from the United States and the United Kingdom of such differentials whereby, relative to White individuals, people of African-Caribbean (Black), Latinx, and South Asian origin experience the greatest burden of infection with severe acute respiratory syndrome coronavirus 2 (SARS-CoV-2)—the virus that causes COVID-19—and hospitalization for, and mortality from, the disease (4, 5).

Understanding how these ethnic variations in COVID-19 are embodied is central to the process of disease prevention. Individuals from different ethnic backgrounds differ in health behaviors, body composition, comorbidities, immune profiles, and socioeconomic circumstances, among other characteristics (6). The best evidence of ethnic differentials in COVID-19 largely draws on observational studies generated from electronic health records where potential explanatory or mediating factors, aside from socioeconomic status and somatic morbidities, are rarely captured (4, 5). Thus, the role of mental health (7), lifestyle factors (e.g., body mass index, alcohol intake) (8), and physiological indices (e.g., systemic inflammation) (9, 10) is untested in this context.

Using data from the UK Biobank, a field-based prospective cohort study, we have shown that people of South Asian and particularly African-Caribbean (Black) heritage experienced a markedly elevated risk of a diagnosis of severe COVID-19, and up to half of these differentials could be explained by socioeconomic status and lifestyle indices (11). In that study, hospitalization for COVID-19 was the outcome of interest. As the pandemic has unfolded, a sufficiently high number of deaths from the disease have accumulated in this cohort to allow us to test these original results with new data.

## METHODS

The UK Biobank is a prospective cohort study, the sampling and procedures of which have been well described (12, 13). Baseline data collection took place between 2006 and 2010 across 22 research assessment centers in the United Kingdom, giving rise to a sample of 502,655 people aged 40–69 years (response 5.5%). Ethical approval was granted by the North-West Multi-centre Research Ethics Committee, and the research was carried out in accordance with the Declaration of Helsinki of the World Medical Association; participants gave written consent.

### Assessment of ethnicity

Data on ethnicity and all covariates used in the present analyses were collected at baseline. Using similar enquiries to those from the 2001 (14) and 2011 (15) UK census, ethnicity was self-classified as White (British, Irish, any other White background); Asian or Asian British (Indian, Pakistani, Bangladeshi, any other South Asian background); Black or Black British (Caribbean, African, any other Black background); mixed; Chinese; or “other” (11). With a low number of COVID-19 deaths occurring in the latter 3 categories owing to the low denominator, these were collapsed into a single “other” group.

### Assessment of covariates

In the present study, we included those covariates shown to be associated with COVID-19 hospitalization in prior analyses (11). Individual socioeconomic status was captured using educational qualifications (university degree, other qualifications, no qualifications). Occupational classification was also available but in a subgroup of participants and based on current job, from which we derived 2 categories: nonmanual (managerial positions, technical, administrative) and manual (sales and customer service; process, plant, and machine operatives). The Townsend index of neighborhood deprivation, a group-level indicator of poverty, is based on national census data, with each participant assigned a score corresponding to the postcode of home address; higher values denote greater disadvantage. The number of people in the household of the study member was also recorded (living alone, 2 people, 3 people, 4 people or more) (16). Levels of cigarette smoking (never, former, current) and alcohol consumption (never, special occasions, 1–3 times/month, 1–2 times week, 3–4 times/week, daily) were assessed using standard enquiries; and height, weight, and circumferences of waist and hip were measured using standard protocols (17). Vascular or heart problems, diabetes, and chronic bronchitis were assessed based on self-reported physician diagnosis, and hypertension was defined as systolic/diastolic blood pressure ≥140/90 mm Hg and/or self-reported use of antihypertensive medication (10). Study members were also asked whether they had ever been under the care of a psychiatrist for any mental health problem (7). Available for a subgroup, white blood cell count (a marker of inflammation), glycated hemoglobin, and high-density lipoprotein cholesterol concentrations were based on assays of nonfasting venous blood.

### Ascertainment of mortality ascribed to COVID-19

Participants were linked to long-standing national mortality records in which death from COVID-19, our outcome of interest, was denoted by the *International Classification of Diseases*, *Tenth Revision*, emergency code U07.1 (COVID-19, virus identified). Deaths were ascertained between March 5, 2020, and the end of follow-up, on January 24, 2021.

### Statistical analyses

To summarize the association of mortality with ethnicity we used logistic regression to compute odds ratios (ORs) with accompanying 95% confidence intervals (CIs). With COVID-19 deaths occurring over a short period and being rare in the present study, ORs very closely resemble hazard ratios as computed using Cox regression analyses. We initially provide age- and sex-adjusted odds ratios, the most basic model and therefore our comparator. We then explored the impact of controlling for individual covariates by making separate (nonaccumulative) adjustment for social factors, lifestyle factors, comorbidities, and biomarkers. Percentage change in effect estimates following statistical control was calculated as: 100 × (β_complex adjustment_ – β_basic adjustment_) / β_basic adjustment_ where basic adjustment was control for age and sex only, and complex adjustment was the addition of further covariates (18). We also present results where all covariates were imputed using chain equations.

**Table 1:** Age- and Sex-Adjusted Odds Ratios for the Association of Ethnicity and Baseline Covariates (2006–2010) With Coronavirus Disease 2019 Mortality (2020–2021), United Kingdom

**Baseline Characteristic**	**Total No.**	**No. of Deaths**	**OR** ^**a**^	**95% CI**	***P* Value**
Ethnicity					
White	426,265	650	1.00	Referent	
Black	6,816	28	4.81	3.28, 7.05	<0.001
South Asian	7,839	19	2.05	1.30, 3.25	0.002
Other	7,774	8	1.19	0.59, 2.40	0.63
Demographic factors					
Age, per 1-year increase			1.15	1.14, 1.17	<0.001
Male sex			2.27	1.94, 2.65	<0.001
Social factors					
Education, high school vs. university			1.92	1.58, 2.33	<0.001
Occupation, manual vs. nonmanual			1.99	1.57, 2.52	<0.001
Household size, living alone vs. ≥2 people			1.98	1.67, 2.35	<0.001
Area-based deprivation index, quintile 5 (highest) vs. 1			2.87	2.28, 3.62	<0.001
Lifestyle factors					
Alcohol, never vs. daily			2.78	2.14, 3.61	<0.001
Cigarette smoking, current vs. never			2.25	1.78, 2.85	<0.001
Body mass index^b^, per unit increase			1.11	1.09, 1.12	<0.001
Waist-to-hip ratio, per 0.1 increase			2.03	1.84, 2.24	<0.001
Comorbidities					
Hypertension, yes vs. no			1.58	1.31, 1.91	<0.001
Cardiovascular disease, yes vs. no			2.25	1.85, 2.73	<0.001
Chronic bronchitis, yes vs. no			1.48	0.93, 2.33	0.10
Diabetes, yes vs. no			3.15	2.60, 3.82	<0.001
Consultation with a psychiatrist, yes vs. no			1.60	1.30, 1.96	<0.001
Biomarkers					
White blood cell count, per 1-log-10^9^/L increase			3.26	2.50, 4.25	<0.001
High-density lipoprotein, per 1-mmol/L increase			0.43	0.33, 0.56	<0.001
HbA1c, per 1-log-mmol/mol increase			7.75	5.51, 10.90	<0.001

Abbreviations: CI, confidence interval; HbA1c, glycated hemoglobin; OR, odds ratio.

^a^ ORs are expressed per category, or per standard-deviation increase for continuous variables. Analyses for occupational classification (*n* = 322,353) and biomarkers (*n* = 358,820) are based on subgroups of study members. All other analyses are based on the full cohort (*n* = 448,664). ORs for age and sex are mutually adjusted.

^b^ Weight (kg)/height (m)^2^.

## RESULTS

Our analytical sample comprised 448,664 individuals (248,820 women). Compared with White study members, at baseline, people from the ethnic minority groups were slightly younger and markedly more likely to live in higher-occupancy households, reside in poorer neighborhoods, work in manual occupations, and have diabetes (Web Table 1, available at https://doi.org/10.1093/aje/kwab237). People from South Asian and other backgrounds were, however, more likely to have experience of higher education than White and Black individuals. Black people had among the lowest prevalence of chronic bronchitis and mental health problems but the highest burden of hypertension.

Mortality surveillance in the analytical sample gave rise to data on 705 deaths from COVID-19 (650 in White participants, 28 in Blacks, 19 in South Asians, and 8 in those from other ethnic groups). In Table 1, we show the age- and sex-adjusted relationships of the above covariates plus ethnicity with the risk of death from COVID-19. Unfavorable levels of all 18 covariates were related to a higher risk of death in minimally adjusted analyses; only the point estimate for chronic bronchitis, while elevated, did not achieve statistical significance at conventional levels. For instance, there was a raised risk of COVID-19 death in people from disadvantaged socioeconomic background, those living alone, those with extant illness at baseline, and those with a higher white blood cell count. While people with less-healthy lifestyle choices typically experienced higher risk, the daily consumption of alcohol seemed to confer some protection.

**Figure 1:**
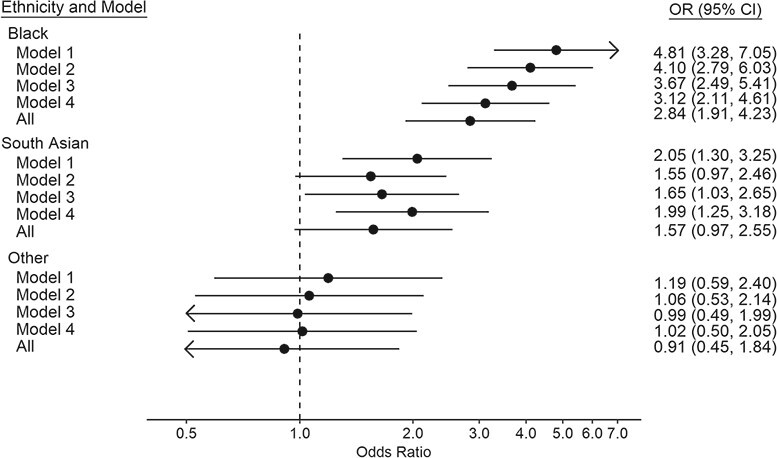
Odds ratios (ORs) for the association between ethnicity (2006–2010) and coronavirus disease 2019 mortality (2020–2021), United Kingdom. Covariates included in each model correspond to those described in Table 1. For the Black participants group, attenuation of regression coefficients was: 28% after controlling for social factors; 17% for lifestyle; 10% for comorbidity; and 34% for all covariates combined. For the South Asian group: 4% after controlling for social factors; 30% for lifestyle; 39% for comorbidities; and 37% for all covariates combined. For the “other” ethnic group: 91% after controlling for social factors; 108% for lifestyle; 66% for comorbidities; and 154% for all covariates combined. CI, confidence interval.

As depicted in Table 1 and Figure 1, relative to White participants, Black study members experienced approximately 5 times the risk of COVID-19 mortality (age- and sex-adjusted OR = 4.81, 95% CI: 3.28, 7.05), while there was approximately a doubling in the South Asian group (OR = 2.05, 95% CI: 1.30, 3.25). There was evidence of a lack of precision in some of these analyses as evidenced by the breadth of the confidence intervals. We explored the impact of individual covariates by making separate (nonaccumulative) adjustment for social factors, lifestyle factors, and comorbidities (Figure 1). In Black participants, relative to the regression coefficients in the age- and sex-adjusted analyses, in separate adjustment, we found that social factors had the largest impact (OR = 3.12, 95% CI: 2.11, 4.61; 28% attenuation), whereas in people of South Asian backgrounds, it was comorbidities (OR = 1.55, 95% CI: 0.97, 2.46; 39% attenuation). Collectively, these covariates accounted for around one-third of the disparity in COVID-19 deaths for Black individuals (OR = 2.84, 95% CI: 1.91, 4.23; 34% attenuation), as they did for South Asian study members (OR = 1.57, 95% CI: 0.97, 2.55; 37% attenuation).

The fact that, despite statistical control for an array of variables, there remained a marked residual risk of death from COVID-19 in ethnic minority groups implicates other risk indices. Biological indices including high-density lipoprotein cholesterol, glycated hemoglobin, and white blood cell count, associated with COVID-19 deaths in the present data set, were available for 358,820 people among whom there were 578 COVID-19 deaths. Adding these variables to the comparator model yielded marked attenuation (OR = 1.35, 95% CI: 0.79, 2.32; 54% attenuation) for South Asian study members but not for Black individuals (OR = 4.69, 95% CI: 2.93, 7.53; 2% attenuation) (Web Table 2).

It is plausible that people from ethnic minority groups are more likely to be in service industry employment that requires them to have a person- or patient-facing role, thus potentially placing them at elevated risk of infection. In analyses of the subgroup with data on job title (*n* = 322,353) there was a total of 328 deaths (Web Table 3). The original raised risk in Black individuals in the main analyses after adjustment for social factors (OR = 3.12, 95% CI: 2.11, 4.61) was elevated slightly when occupation was added to the model (OR = 3.96, 95% CI: 2.46, 6.36); again, statistical precision was modest owing to the small numbers of COVID-19 fatalities in this and other minority groups. Last, on imputing covariates (Web Table 4), a similar pattern of association was observed to that apparent in the main analyses, with an age- and sex-adjusted odds ratio for Black individuals of 3.99 (95% CI: 2.74, 5.80), which was attenuated by 31% after multiple adjustment including occupation and biomarkers, to 2.58 (95% CI: 1.74, 3.84). In corresponding analyses for people from South Asian background, attenuation after the same statistical control was 47%.

## DISCUSSION

Our main finding was that, despite statistical control for social factors, lifestyle indices, biological factors, and comorbidities, there remained a markedly raised risk of COVID-19 mortality in people of African-Caribbean and South Asian origin in the United Kingdom. That we were able to replicate known associations with COVID-19 mortality for socioeconomic circumstances, comorbidities, age, and sex apparent in studies from the United States (19), United Kingdom (20), Italy (21), China (22), and Brazil (23) gives us some confidence in the more novel results presented here for ethnicity.

The marked postadjustment excess risk of COVID-19 in Black and South Asian study members suggests that unmeasured and/or unknown risk factors have a role (6). While the present data set is reasonably well-characterized for environmental factors, we do not have data on, for instance, life-course socioeconomic position or racial discrimination. Although understudied, racial discrimination appears to have an influence on selected health outcomes, most consistently mental health (24) and, of more relevance to the present study, respiratory conditions such as adult-onset asthma (25). While vigorously advanced in some quarters as having a causative role in the current pandemic (26–29), to the best of our knowledge, such links are untested empirically, rendering moot its role.

### Comparison with existing studies

Although less well-examined owing to its lower impact relative to the present pandemic, H1N1 revealed similar ethnic differentials to those reported herein (1, 2). The Spanish influenza of 1918 was perhaps an exception to the typical picture of a greater burden in minority groups: Rates of hospitalization and death were in fact seemingly lower in people of Black ethnic origin relative to Whites in the United States (30)—the only year in the 20th century when being of Black origin appeared to confer some protection against death from influenza. In the current pandemic, the present findings of ethnic disparities are supported by observations made on populations from the United States and the United Kingdom (4, 5). As discussed, while in-depth examination of the causes of these inequalities is rare owing to an absence of higher-resolution data in most studies, effects seem to survive adjustment for extant morbidity and, when available, markers of poverty (4, 5) (31–34). Partial attenuation by comorbidity, which to our knowledge featured mental illness for the first time (7), was also seen herein, with a further diminution in risk offered by lifestyle and social factors, which confirms our earlier work on hospitalizations for the disease (11). Unlike the present analyses featuring death as the outcome of interest, in that study (11) we used a record of a positive inpatient test for COVID-19 as our outcome of interest. While this was assumed to be an indicator of disease severity—only serious cases are hospitalized in the United Kingdom, which operates under a single, national health service—it is nonetheless likely that, after routine hospital-wide testing, some patients being treated for unrelated conditions were positive but asymptomatic for COVID-19. Our results here for death from the disease corroborates these earlier findings, however (11).

### Study strengths and weaknesses

The strengths of the study include the well-characterized nature of the study members and the full coverage of the population for cause of death from COVID-19. Our work is of course not without its weaknesses. Although the present cohort is large, there were too few deaths in selected ethnic groups—people from East Asian or mixed backgrounds, for instance—to facilitate analyses. Also, while ethnicity itself is stable over-time—UK data reveal that only 4% of census participants chose a different ethnic group a decade after their first declaration (35)—other baseline data are more likely to be time-varying in the period between study induction in the UK Biobank and the present pandemic, in particular for comorbidity. This is a perennial issue in cohort studies and one we were able to investigate using data from a resurvey that took place around 8 years after baseline examination in a subsample of approximately 30,000 people. Analyses revealed moderate to high stability for some covariates, including education (*r* = 0.86, *P* < 0.001) and body mass index (*r* = 0.90, *P* < 0.001), whereas the magnitude was somewhat lower for diabetes (*r* = 0.63, *P* < 0.001), serious mental illness (*r* = 0.64, *P* < 0.001), and cigarette smoking (*r* = 0.60, *P* < 0.001, *n* = 31,037).

### Generalizability of the present findings

With the present sample not being representative of the general UK population, death rates from leading causes and the prevalence of reported risk factors are known to be underestimates of those apparent in less-select groups (13); the same is likely to be the case for COVID-19 cases. This notwithstanding, for the following reasons, there is evidence that risk-factor associations, including those presented herein for ethnicity, are externally valid (13). First, the ethnic distribution in the UK Biobank is similar to UK 2001 and 2011 census data (Web Table 5). Second, relative to White Europeans, we found that South Asians have a markedly higher prevalence of diabetes and less favorable waist-to-hip ratio, whereas the greatest burden of hypertension was in Black and White participants (Web Table 1). These observations have been made across multiple studies **(**36–38). Third, consistently higher rates of coronary heart disease in South Asians (the reverse in Black individuals), and a lower risk of cancer have been reported (36, 38–40). In analyses of data from the present study, we found this pattern of association (Web Table 6). Last, as described, in keeping with systematic reviews of ethnicity and COVID-19 (4, 5), we have shown an increased risk of hospitalization for COVID-19 among minority groups in the United Kingdom (11). Taken together then, we regard the present results from the UK Biobank to be generalizable.

In conclusion, in this well-characterized prospective cohort study, based on conventional risk factors, we were only able to partially understand how ethnic disparities in COVID-19 were embodied. Subsequent research should target additional factors uncaptured herein, including life-course socioeconomic position and racial discrimination.

## Supplementary Material

Web_Material_kwab237
