# Supplementary material for: Explaining Ethnic Differentials in COVID-19 Mortality: A Cohort Study
Source: Am J Epidemiol. 2021 Sep 29;191(2):275–81. doi: 10.1093/aje/kwab237 (PMC8513410; doi:10.1093/aje/kwab237)
Supplement: Web_Material_kwab237 [file web_material_kwab237.pdf]

Web Material

Explaining Ethnic Differentials in Coronavirus Disease 2019 Mortality: A Cohort Study

G. David Batty (david.batty@ucl.ac.uk)
Department of Epidemiology and Public Health, University College London, UK

Bamba Gaye (bamba.gaye@innserm.fr)
Paris Cardiovascular Research Center-INSERM U970, France

Catharine R. Gale (crg@mrc.soton.ac.uk)
MRC Lifecourse Epidemiology Unit, University of Southampton, UK
Lothian Birth Cohorts, Department of Psychology, University of Edinburgh, UK

Mark Hamer (m.hamer@ucl.ac.uk)
Division of Surgery and Interventional Sciences, University College London, London

and

Camille Lassale (classale@imim.es)
Hospital del Mar Medical Research Institute, Barcelona, Spain
CIBER of Pathophysiology of Obesity and Nutrition, Madrid, Spain

Web Table 1. Baseline characteristics of the analytical sample according to ethnic group (2006-10)

Web Table 2. Odds ratios (95% confidence intervals) for the association of ethnicity (2006-10) with COVID-19 mortality (2020-21) – sub-sample with biomarker data (N=358,820; 578 deaths)

Web Table 3. Odds ratios (95% confidence intervals) for the association of ethnicity (2006-10) with COVID-19 mortality (2020-21) in a sub-sample with occupational data (N=322,353; 328 deaths)

Web Table 4. Odds ratios (95% confidence intervals) for the association of ethnicity (2006-10) with COVID-19 (2020-21) – based on 2 imputed datasets (N= 470,665; 781 deaths)

Web Table 5. Comparison of the self-declared ethnic groups in UK Biobank (2006–2010) with UK censuses in 2001 and 2011 – all comparisons are for people aged 40–69 years at assessment

Web Table 6. Age- and sex-adjusted hazard ratios (95% confidence intervals) for the relation of ethnicity with mortality outcomes in UK Biobank

Web Table 1. Baseline characteristics of the analytical sample according to ethnic group (2006-10)

	Black	South Asian	Other	White
Number	6,816	7,839	7,744	426,265
Women, N (%)	4012 (58.9)	3741 (47.7)	4695 (60.6)	236372 (55.5)
Age, years, mean (SD)	51.7 (7.9)	53.1 (8.4)	52.1 (7.8)	56.4 (8.0)
Higher education, N (%)	2371 (34.8)	3382 (43.1)	3627 (46.8)	141140 (33.1)
Household ≥4 people, N (%)	2195 (32.2)	3991 (50.9)	2455 (31.7)	78889 (18.5)
High neighbourhood deprivation, N (%)	4370 (64.1)	2873 (36.7)	3371 (43.5)	78997 (18.5)
Manual occupation, N (%)	1597 (31.2)	1527 (26.7)	1280 (22.8)	54710 (17.9)
Alcohol intake daily, N (%)	451 (6.6)	546 (7)	777 (10)	89829 (21.1)
Current smoking, N (%)	795 (11.7)	704 (9.0)	1047 (13.5)	41163 (9.7)
Hypertension, N (%)	4200 (61.6)	4327 (55.2)	3769 (48.7)	246517 (57.8)
Diabetes, N (%)	700 (10.3)	1239 (15.8)	588 (7.6)	18342 (4.3)
Cardiovascular disease, N (%)	280 (4.1)	577 (7.4)	284 (3.7)	21422 (5)
Chronic bronchitis, N (%)	38 (0.6)	70 (0.9)	64 (0.8)	5622 (1.3)
Seen a psychiatrist, N (%)	565 (8.3)	760 (9.7)	967 (12.5)	48070 (11.3)
BMI, kg/m,^{2} mean (SD)	29.6 (5.4)	27.1 (4.4)	26.0 (5.0)	27.3 (4.7)
Waist to hip ratio, mean (SD)	0.87 (0.08)	0.90 (0.08)	0.86 (0.08)	0.87 (0.09)
White blood cells, 10^9/L, mean (SD)	5.78 (1.66)	7.30 (1.75)	6.76 (1.93)	6.85 (1.96)
HbA1c, mmol/mol, mean (SD)	39.2 (9.9)	40.1 (9.8)	37.3 (8.0)	35.8 (6.2)
HDL-cholesterol, mmol/L, mean (SD)	1.44 (0.36)	1.27 (0.33)	1.42 (0.38)	1.46 (0.38)

Web Table 2. Odds ratios (95% confidence intervals) for the association of ethnicity (2006-10) with COVID-19 mortality (2020-21) – sub-sample with biomarker data (N=358,820; 578 deaths)

**Ethnicity** (no. deaths/no people)	**Adjustment**	**Odds ratio**	**95% confidence interval**	**P-value**	**Attenuation (%)**
Black (19/4,445)	Age- and sex-adjusted	4.87	3.07, 7.73	<0.001	
	+ Social factors	3.07	1.91, 4.91	<0.001	-29.2
	+ Lifestyle factors	3.69	2.31, 5.90	<0.001	-17.5
	+ Comorbidities	4.27	2.68, 6.80	<0.001	-8.3
	+ Biomarkers	4.69	2.93, 7.53	<0.001	-2.3
	Multiply-adjusted	3.10	1.91, 5.05	<0.001	-28.5
South Asian (14/6,005)	Age- and sex-adjusted	1.92	1.13, 3.28	0.08	
	+ Social factors	1.82	1.06, 3.14	0.17	-8.0
	+ Lifestyle factors	1.52	0.88, 2.62	0.26	-36.4
	+ Comorbidities	1.48	0.86, 2.53	0.20	-40.3
	+ Biomarkers	1.35	0.79, 2.32	0.32	-53.6
	Multiply-adjusted	1.36	0.78, 2.39	0.53	-52.8
Other (7/5,943)	Age- and sex-adjusted	1.29	0.61, 2.73	0.43	
	+ Social factors	1.10	0.52, 2.33	0.67	-62.8
	+ Lifestyle factors	1.06	0.50, 2.25	0.64	-76.9
	+ Comorbidities	1.17	0.55, 2.47	0.51	-39.4
	+ Biomarkers	1.14	0.54, 2.41	0.55	-49.2
	Multiply-adjusted	0.98	0.46, 2.08	0.83	-107.9

The reference category is White ethnicity (538 deaths in 342,427 people). Socioeconomic status: education, area deprivation index, household size; Lifestyle factors: alcohol consumption, cigarette smoking, body mass index, waist to hip ratio; Comorbidities: hypertension, diabetes, cardiovascular disease, chronic bronchitis, consultation with a psychiatrist; Biomarkers: HDL-cholesterol, white blood cell count, glycated haemoglobin.

Web Table 3. Odds ratios (95% confidence intervals) for the association of ethnicity (2006-10) with COVID-19 mortality (2020-21) in a sub-sample with occupational data (N=322,353; 328 deaths)

**Ethnicity** (no. deaths/no people)	**Adjustment**	**Odds ratio**	**95% confidence interval**	**P-value**	**Attenuation (%)**
Black (20/5,099)	Age- and sex-adjusted	6.46	4.09, 10.20	<0.001	
	+ Social factors plus occupation	3.96	2.46, 6.36	<0.001	-26.3
	+ Lifestyle factors	4.53	2.83, 7.25	<0.001	-19.0
	+ Comorbidities	5.57	3.51, 8.85	<0.001	-7.9
	Multiply-adjusted	3.30	2.02, 5.37	<0.001	-36.1
South Asian (8/5,709)	Age- and sex-adjusted	1.68	0.83, 3.40	0.15	
	+ Social factors plus occupation	1.51	0.74, 3.08	0.26	-21.2
	+ Lifestyle factors	1.18	0.57, 2.42	0.66	-68.6
	+ Comorbidities	1.34	0.66, 2.72	0.42	-44.1
	Multiply-adjusted	1.09	0.52, 2.28	0.82	-83.6
Other (4/5,620)	Age- and sex-adjusted	1.15	0.43, 3.09	0.78	
	+ Social factors plus occupation	0.95	0.35, 2.55	0.91	-139.0
	+ Lifestyle factors	0.90	0.33, 2.44	0.84	-173.8
	+ Comorbidities	1.04	0.39, 2.81	0.93	-69.2
	Multiply-adjusted	0.82	0.30, 2.22	0.70	-242.0

The reference category is White ethnicity (296 deaths in 305,925 people). Socioeconomic status: education, area deprivation index, household size, occupation; Lifestyle factors: alcohol consumption, cigarette smoking, body mass index, waist to hip ratio; Comorbidities: hypertension, diabetes, cardiovascular disease, chronic bronchitis, consultation with a psychiatrist.

Web Table 4. Odds ratios (95% confidence intervals) for the association of ethnicity (2006-10) with COVID-19 (2020-21) – based on 2 imputed datasets (N= 470,665; 781 deaths)

**Ethnicity** (no. deaths/no people)	**Adjustment**	**Odds ratio**	**95% confidence interval**	**P-value**	**Attenuation (%)**
Black (29/7,779)	Age- and sex-adjusted	3.99	2.74, 5.80	<0.001	
	+ Social factors	2.66	1.81, 3.90	<0.001	-29.3
	+ Social factors plus occupation	2.52	1.71, 3.69	<0.001	-33.3
	+ Lifestyle factors	3.06	2.09, 4.47	<0.001	-19.3
	+ Comorbidities	3.39	2.32, 4.95	<0.001	-11.8
	+ Biomarkers	3.84	2.62, 5.62	<0.001	-2.8
	Multiply-adjusted without biomarkers	2.35	1.59, 3.47	<0.001	-38.3
	Multiply-adjusted	2.58	1.74, 3.84	<0.001	-31.4
South Asian (22/9,489)	Age- and sex-adjusted	1.87	1.22, 2.87	0.004	
	+ Social factors	1.81	1.17, 2.80	0.01	-5.7
	+ Social factors plus occupation	1.74	1.12, 2.70	0.01	-12.0
	+ Lifestyle factors	1.53	0.99, 2.38	0.06	-32.0
	+ Comorbidities	1.40	0.91, 2.16	0.12	-46.1
	+ Biomarkers	1.33	0.86, 2.04	0.20	-55.0
	Multiply-adjusted without biomarkers	1.44	0.91, 2.27	0.12	-42.2
	Multiply-adjusted	1.40	0.88, 2.20	0.15	-46.9
Other (10/8,756)	Age- and sex-adjusted	1.23	0.66, 2.30	0.52	
	+ Social factors	1.06	0.56, 1.98	0.86	-72.7
	+ Social factors plus occupation	1.03	0.55, 1.93	0.93	-86.8
	+ Lifestyle factors	1.02	0.54, 1.91	0.95	-90.1
	+ Comorbidities	1.09	0.58, 2.05	0.78	-56.7
	+ Biomarkers	1.08	0.58, 2.03	0.80	-60.7
	Multiply-adjusted without biomarkers	0.95	0.51, 1.79	0.88	-124.2
	Multiply-adjusted	0.96	0.51, 1.81	0.91	-118.5

The reference category is White ethnicity (720 deaths in 444,641). Socioeconomic status: education, area deprivation index, household size; Lifestyle factors: alcohol consumption, cigarette smoking, body mass index, waist to hip ratio; Comorbidities: hypertension, diabetes, cardiovascular disease, chronic bronchitis, consultation with a psychiatrist; Biomarkers: HDL-cholesterol, white blood cell count, glycated haemoglobin

Web Table 5. Comparison of the self-declared ethnic groups in UK Biobank (2006–2010) with UK censuses in 2001 and 2011 – all comparisons are for people aged 40–69 years at assessment

Ethnicity groups in present analyses	UK Biobank (n = 499,877)	2001 UK Census[23] (n = 20,198,307)	2011 UK Census[24] (n = 23,146,612)
White	94.6 (472,837)	94.5 (19,085,322)	91.3 (21,133,317)
Black/Black British	1.6 (8,066)	1.5 (302,073)	2.4 (565,777)
South Asian	1.6 (8,024)	2.5 (519,566)	3.2 (757,423)
Other	2.2 (10,950)	1.5 (291,346)	3.1 (690,095)

Results are % (N)

Web Table 6. Age- and sex-adjusted hazard ratios (95% confidence intervals) for the relation of ethnicity with mortality outcomes in UK Biobank

		White	Other	South Asian	Black
**COVID-19***	N cases / N risk	650/426265	8/7744	19/7839	28/6816
	Odds ratios (95% CI)	1.0 (ref)	1.19 (0.59, 2.40)	2.05 (1.30, 3.25)	4.81 (3.28, 7.05)
**All deaths**	N cases / N risk	32483/472729	374/9091	469/9882	342/8061
	Hazard ratios (95% CI)	1.0	0.90 (0.81, 1.00)	0.86 (0.78, 0.94)	0.93 (0.84, 1.04)
**CHD**	N cases / N risk	5293/472729	56/9091	169/9882	42/8061
	Hazard ratios (95% CI)	1.0	0.91 (0.61, 1.19)	1.82 (1.57, 2.13)	0.77 (0.57, 1.05)
**Stroke**	N cases / N risk	1973/472729	23/9091	33/9882	28/8061
	Hazard ratios (95% CI)	1.0	1.01 (0.69, 1.52)	1.07 (0.76, 1.51)	1.38 (0.95, 2.01)
**All cancers**	N cases / N risk	16609/472729	206/9091	174/9882	159/8061
	Hazard ratios (95% CI)	1.0	0.88 (0.76, 1.01)	0.59 (0.50, 0.68)	0.77 (0.66, 0.91)

*Results from present manuscript. Deaths from COVID-19 are from 5th March 2020, to 24th January, 2021; deaths from other causes are from 10th May 2006 to 18th December 2020.
