# Supplementary material for: Explaining ethnic differentials in COVID-19 mortality: cohort study
Source: Am J Epidemiol. 2021 Sep 29:kwab237. doi: 10.1093/aje/kwab237 (PMC8513410; doi:10.1093/aje/kwab237)
Supplement: Supplementary file 1 [file Web_Material_kwab237.docx]

**Web material** 

**Explaining ethnic differentials in COVID-19 mortality: cohort study**

G. David Batty (david.batty@ucl.ac.uk)

Department of Epidemiology and Public Health, University College London, UK 

Bamba Gaye (bamba.gaye@innserm.fr)

Paris Cardiovascular Research Center-INSERM U970, France

Catharine R. Gale (crg@mrc.soton.ac.uk)

MRC Lifecourse Epidemiology Unit, University of Southampton, UK

Lothian Birth Cohorts, Department of Psychology, University of Edinburgh, UK

Mark Hamer (m.hamer@ucl.ac.uk)

Division of Surgery and Interventional Sciences, University College London, London

and

Camille Lassale (classale@imim.es)

Hospital del Mar Medical Research Institute, Barcelona, Spain

CIBER of Pathophysiology of Obesity and Nutrition, Madrid, Spain

*Web Table 1.  Baseline characteristics of the analytical sample*

*according to ethnic group (2006-10)*

*Web Table 2.  Odds ratios (95% confidence intervals) for* 

*the association of ethnicity (2006-10) with COVID-19 mortality (2020-21) –* 

*sub-sample with biomarker data (N=358,820; 578 deaths)*

*Web Table 3.  Odds ratios (95% confidence intervals) for* 

*the association of ethnicity (2006-10) with COVID-19 mortality (2020-21) in a sub-sample with occupational data (N=322,353; 328 deaths)*

*Web Table 4. Odds ratios (95% confidence intervals) for* 

*the association of ethnicity (2006-10) with COVID-19 (2020-21) –* 

*based on 2 imputed datasets (N= 470,665; 781 deaths)*

*Web Table 5.  Comparison of the self-declared ethnic groups in UK Biobank (2006–2010) with UK censuses in 2001 and 2011 – all comparisons are for people aged 40–69 years at assessment*

*Web Table 6.  Age- and sex-adjusted hazard ratios (95% confidence intervals) for the* 

*relation of ethnicity with mortality outcomes in UK Biobank*

**Web Table 1.  Baseline characteristics of the analytical sample**

**according to ethnic group (2006-10)**

|  | **Black** | **South Asian** | **Other** | **White** |
| --- | --- | --- | --- | --- |
| Number | 6,816 | 7,839 | 7,744 | 426,265 |
| Women, N (%) | 4012 (58.9) | 3741 (47.7) | 4695 (60.6) | 236372 (55.5) |
| Age, years, mean (SD) | 51.7 (7.9) | 53.1 (8.4) | 52.1 (7.8) | 56.4 (8.0) |
| Higher education, N (%) | 2371 (34.8) | 3382 (43.1) | 3627 (46.8) | 141140 (33.1) |
| Household ≥4 people, N (%) | 2195 (32.2) | 3991 (50.9) | 2455 (31.7) | 78889 (18.5) |
| High neighbourhood deprivation, N (%) | 4370 (64.1) | 2873 (36.7) | 3371 (43.5) | 78997 (18.5) |
| Manual occupation, N (%) | 1597 (31.2) | 1527 (26.7) | 1280 (22.8) | 54710 (17.9) |
| Alcohol intake daily, N (%) | 451 (6.6) | 546 (7) | 777 (10) | 89829 (21.1) |
| Current smoking, N (%) | 795 (11.7) | 704 (9.0) | 1047 (13.5) | 41163 (9.7) |
| Hypertension, N (%) | 4200 (61.6) | 4327 (55.2) | 3769 (48.7) | 246517 (57.8) |
| Diabetes, N (%) | 700 (10.3) | 1239 (15.8) | 588 (7.6) | 18342 (4.3) |
| Cardiovascular disease, N (%) | 280 (4.1) | 577 (7.4) | 284 (3.7) | 21422 (5) |
| Chronic bronchitis, N (%) | 38 (0.6) | 70 (0.9) | 64 (0.8) | 5622 (1.3) |
| Seen a psychiatrist, N (%) | 565 (8.3) | 760 (9.7) | 967 (12.5) | 48070 (11.3) |
| BMI, kg/m,^2^ mean (SD) | 29.6 (5.4) | 27.1 (4.4) | 26.0 (5.0) | 27.3 (4.7) |
| Waist to hip ratio, mean (SD) | 0.87 (0.08) | 0.90 (0.08) | 0.86 (0.08) | 0.87 (0.09) |
| White blood cells, 10^9^/L, mean (SD) | 5.78 (1.66) | 7.30 (1.75) | 6.76 (1.93) | 6.85 (1.96) |
| HbA1c, mmol/mol, mean (SD) | 39.2 (9.9) | 40.1 (9.8) | 37.3 (8.0) | 35.8 (6.2) |
| HDL-cholesterol, mmol/L, mean (SD) | 1.44 (0.36) | 1.27 (0.33) | 1.42 (0.38) | 1.46 (0.38) |

**Web Table 2.  Odds ratios (95% confidence intervals) for** 

**the association of ethnicity (2006-10) with COVID-19 mortality (2020-21) –** 

**sub-sample with biomarker data (N=**358,820; 578 deaths)

| **Ethnicity** (no. deaths/no people) | **Adjustment** | **Odds ratio** | **95% confidence interval** | **P-value** | **Attenuation (%)** |
| --- | --- | --- | --- | --- | --- |
| Black (19/4,445) | Age- and sex-adjusted | 4.87 | 3.07, 7.73 | <0.001 |  |
|  | + Social factors | 3.07 | 1.91, 4.91 | <0.001 | -29.2 |
|  | + Lifestyle factors | 3.69 | 2.31, 5.90 | <0.001 | -17.5 |
|  | + Comorbidities | 4.27 | 2.68, 6.80 | <0.001 | -8.3 |
|  | + Biomarkers | 4.69 | 2.93, 7.53 | <0.001 | -2.3 |
|  | Multiply-adjusted | 3.10 | 1.91, 5.05 | <0.001 | -28.5 |
| South Asian (14/6,005) | Age- and sex-adjusted | 1.92 | 1.13, 3.28 | 0.08 |  |
|  | + Social factors | 1.82 | 1.06, 3.14 | 0.17 | -8.0 |
|  | + Lifestyle factors | 1.52 | 0.88, 2.62 | 0.26 | -36.4 |
|  | + Comorbidities | 1.48 | 0.86, 2.53 | 0.20 | -40.3 |
|  | + Biomarkers | 1.35 | 0.79, 2.32 | 0.32 | -53.6 |
|  | Multiply-adjusted | 1.36 | 0.78, 2.39 | 0.53 | -52.8 |
| Other (7/5,943) | Age- and sex-adjusted | 1.29 | 0.61, 2.73 | 0.43 |  |
|  | + Social factors | 1.10 | 0.52, 2.33 | 0.67 | -62.8 |
|  | + Lifestyle factors | 1.06 | 0.50, 2.25 | 0.64 | -76.9 |
|  | + Comorbidities | 1.17 | 0.55, 2.47 | 0.51 | -39.4 |
|  | + Biomarkers | 1.14 | 0.54, 2.41 | 0.55 | -49.2 |
|  | Multiply-adjusted | 0.98 | 0.46, 2.08 | 0.83 | -107.9 |

The reference category is White ethnicity (538 deaths in 342,427 people).  Socioeconomic status: education, area deprivation index, household size; Lifestyle factors: alcohol consumption, cigarette smoking, body mass index, waist to hip ratio; Comorbidities: hypertension, diabetes, cardiovascular disease, chronic bronchitis, consultation with a psychiatrist; Biomarkers: HDL-cholesterol, white blood cell count, glycated haemoglobin.

**Web Table 3.  Odds ratios (95% confidence intervals) for** 

**the association of ethnicity (2006-10) with COVID-19 mortality (2020-21) in a sub-sample with occupational data (N=**322,353; 328 deaths)

| **Ethnicity** (no. deaths/no people) | **Adjustment** | **Odds ratio** | **95% confidence interval** | **P-value** | **Attenuation (%)** |
| --- | --- | --- | --- | --- | --- |
| Black (20/5,099) | Age- and sex-adjusted | 6.46 | 4.09, 10.20 | <0.001 |  |
|  | + Social factors plus occupation | 3.96 | 2.46, 6.36 | <0.001 | -26.3 |
|  | + Lifestyle factors | 4.53 | 2.83, 7.25 | <0.001 | -19.0 |
|  | + Comorbidities | 5.57 | 3.51, 8.85 | <0.001 | -7.9 |
|  | Multiply-adjusted | 3.30 | 2.02, 5.37 | <0.001 | -36.1 |
| South Asian (8/5,709) | Age- and sex-adjusted | 1.68 | 0.83, 3.40 | 0.15 |  |
|  | + Social factors plus occupation | 1.51 | 0.74, 3.08 | 0.26 | -21.2 |
|  | + Lifestyle factors | 1.18 | 0.57, 2.42 | 0.66 | -68.6 |
|  | + Comorbidities | 1.34 | 0.66, 2.72 | 0.42 | -44.1 |
|  | Multiply-adjusted | 1.09 | 0.52, 2.28 | 0.82 | -83.6 |
| Other (4/5,620) | Age- and sex-adjusted | 1.15 | 0.43, 3.09 | 0.78 |  |
|  | + Social factors plus occupation | 0.95 | 0.35, 2.55 | 0.91 | -139.0 |
|  | + Lifestyle factors | 0.90 | 0.33, 2.44 | 0.84 | -173.8 |
|  | + Comorbidities | 1.04 | 0.39, 2.81 | 0.93 | -69.2 |
|  | Multiply-adjusted | 0.82 | 0.30, 2.22 | 0.70 | -242.0 |

The reference category is White ethnicity (296 deaths in 305,925 people).  Socioeconomic status: education, area deprivation index, household size, occupation; Lifestyle factors: alcohol consumption, cigarette smoking, body mass index, waist to hip ratio; Comorbidities: hypertension, diabetes, cardiovascular disease, chronic bronchitis, consultation with a psychiatrist. 

**Web Table 4.** **Odds ratios (95% confidence intervals) for** 

**the association of ethnicity (2006-10) with COVID-19 (2020-21) –** 

**based on 2 imputed datasets (N=** 470,665; 781 deaths)

| **Ethnicity**  (no. deaths/no people) | **Adjustment** | **Odds ratio** | **95% confidence interval** | **P-value** | **Attenuation (%)** |
| --- | --- | --- | --- | --- | --- |
|  |  |  |  |  |  |
| Black (29/7,779) | Age- and sex-adjusted | 3.99 | 2.74, 5.80 | <0.001 |  |
|  | + Social factors | 2.66 | 1.81, 3.90 | <0.001 | -29.3 |
|  | + Social factors plus occupation | 2.52 | 1.71, 3.69 | <0.001 | -33.3 |
|  | + Lifestyle factors | 3.06 | 2.09, 4.47 | <0.001 | -19.3 |
|  | + Comorbidities | 3.39 | 2.32, 4.95 | <0.001 | -11.8 |
|  | + Biomarkers | 3.84 | 2.62, 5.62 | <0.001 | -2.8 |
|  | Multiply-adjusted without biomarkers | 2.35 | 1.59, 3.47 | <0.001 | -38.3 |
|  | Multiply-adjusted | 2.58 | 1.74, 3.84 | <0.001 | -31.4 |
| South Asian (22/9,489) | Age- and sex-adjusted | 1.87 | 1.22, 2.87 | 0.004 |  |
|  | + Social factors | 1.81 | 1.17, 2.80 | 0.01 | -5.7 |
|  | + Social factors plus occupation | 1.74 | 1.12, 2.70 | 0.01 | -12.0 |
|  | + Lifestyle factors | 1.53 | 0.99, 2.38 | 0.06 | -32.0 |
|  | + Comorbidities | 1.40 | 0.91, 2.16 | 0.12 | -46.1 |
|  | + Biomarkers | 1.33 | 0.86, 2.04 | 0.20 | -55.0 |
|  | Multiply-adjusted without biomarkers | 1.44 | 0.91, 2.27 | 0.12 | -42.2 |
|  | Multiply-adjusted | 1.40 | 0.88, 2.20 | 0.15 | -46.9 |
| Other (10/8,756) | Age- and sex-adjusted | 1.23 | 0.66, 2.30 | 0.52 |  |
|  | + Social factors | 1.06 | 0.56, 1.98 | 0.86 | -72.7 |
|  | + Social factors plus occupation | 1.03 | 0.55, 1.93 | 0.93 | -86.8 |
|  | + Lifestyle factors | 1.02 | 0.54, 1.91 | 0.95 | -90.1 |
|  | + Comorbidities | 1.09 | 0.58, 2.05 | 0.78 | -56.7 |
|  | + Biomarkers | 1.08 | 0.58, 2.03 | 0.80 | -60.7 |
|  | Multiply-adjusted without biomarkers | 0.95 | 0.51, 1.79 | 0.88 | -124.2 |
|  | Multiply-adjusted | 0.96 | 0.51, 1.81 | 0.91 | -118.5 |

The reference category is White ethnicity (720 deaths in 444,641).  Socioeconomic status: education, area deprivation index, household size; Lifestyle factors: alcohol consumption, cigarette smoking, body mass index, waist to hip ratio; Comorbidities: hypertension, diabetes, cardiovascular disease, chronic bronchitis, consultation with a psychiatrist; Biomarkers: HDL-cholesterol, white blood cell count, glycated haemoglobin

**Web Table 5.  Comparison of the self-declared ethnic groups in UK Biobank (2006–2010) with UK censuses in 2001 and 2011 – all comparisons are for people aged 40–69 years at assessment**

| **Ethnicity groups in present analyses** | **UK Biobank** **(n = 499,877)** | **2001 UK Census**^**23**^ **(n = 20,198,307)** | **2011 UK Census**^**24**^ **(n = 23,146,612)** |
| --- | --- | --- | --- |
| White | 94.6 (472,837) | 94.5 (19,085,322) | 91.3 (21,133,317) |
| Black/Black British | 1.6 (8,066) | 1.5 (302,073) | 2.4 (565,777) |
| South Asian | 1.6 (8,024) | 2.5 (519,566) | 3.2 (757,423) |
| Other | 2.2 (10,950) | 1.5 (291,346) | 3.1 (690,095) |

 Results are % (N)

**Web Table 6.  Age- and sex-adjusted hazard ratios (95% confidence intervals) for the** 

**relation of ethnicity with mortality outcomes in UK Biobank**

|  |  | **White** | **Other** | **South Asian** | **Black** |
| --- | --- | --- | --- | --- | --- |
| **COVID-19*** | N cases / N risk | 650/426265 | 8/7744 | 19/7839 | 28/6816 |
|  | Odds ratios (95% CI) | 1.0 (ref) | 1.19 (0.59, 2.40) | 2.05 (1.30, 3.25) | 4.81 (3.28, 7.05) |
| **All deaths** | N cases / N risk | 32483/472729 | 374/9091 | 469/9882 | 342/8061 |
|  | Hazard ratios (95% CI) | 1.0 | 0.90 (0.81, 1.00) | 0.86 (0.78, 0.94) | 0.93 (0.84, 1.04) |
| **CHD** | N cases / N risk | 5293/472729 | 56/9091 | 169/9882 | 42/8061 |
|  | Hazard ratios (95% CI) | 1.0 | 0.91 (0.61, 1.19) | 1.82 (1.57, 2.13) | 0.77 (0.57, 1.05) |
| **Stroke** | N cases / N risk | 1973/472729 | 23/9091 | 33/9882 | 28/8061 |
|  | Hazard ratios (95% CI) | 1.0 | 1.01 (0.69, 1.52) | 1.07 (0.76, 1.51) | 1.38 (0.95, 2.01) |
| **All cancers** | N cases / N risk | 16609/472729 | 206/9091 | 174/9882 | 159/8061 |
|  | Hazard ratios (95% CI) | 1.0 | 0.88 (0.76, 1.01) | 0.59 (0.50, 0.68) | 0.77 (0.66, 0.91) |

*Results from present manuscript.  Deaths from COVID-19 are from 5th March 2020, to 24th January, 2021; deaths from other causes are from 10th May 2006 to 18th December 2020.
